# Degradation of 2,4-dichlorophenoxyacetic acid (2,4-D) and 2,4,5-trichlorophenoxyacetic acid (2,4,5-T) by fungi originating from Vietnam

**DOI:** 10.1007/s10532-022-09982-1

**Published:** 2022-05-02

**Authors:** Thi Lan Anh Nguyen, Anh Thi Ngoc Dao, Ha Thi Cam Dang, Jacco Koekkoek, Abraham Brouwer, Tjalf E. de Boer, Rob J. M. van Spanning

**Affiliations:** 1 Department of Molecular Cell Biology, Vrije Universiteit, De Boelelaan 1108, 1081 HZ Amsterdam, The Netherlands; 2 Institute of Biotechnology, Vietnam Academy of Science and Technology, 18 Hoang Quoc Viet, Cau Giay, Hanoi, Vietnam; 3 Department of Environment and Health, Vrije Universiteit, De Boelelaan 1108, 1081 HZ Amsterdam, The Netherlands; 4 MicroLife Solutions, Science Park 406, 1098 XH Amsterdam, The Netherlands; 5 BioDetection Systems, Science Park 406, 1098 XH Amsterdam, The Netherlands; 6 Department of Ecological Science, Vrije Universiteit, De Boelelaan 1085, 1081 HV Amsterdam, The Netherlands

**Keywords:** Biodegradation, 2,4-Dichlorophenoxyacetic acid, 2,4,5-Trichlorophenoxyacetic acid, Laccase, CYP inhibitors, *Rigidoporus*

## Abstract

Three different fungi were tested for their ability to degrade 2,4-dichlorophenoxyacetic acid and 2,4,5-trichlorophenoxyacetic acid and for the role of laccases and cytochromes P450-type in this process. We studied a white-rot fungus *Rigidoporus* sp. FMD21, which has a high laccase activity, for its efficiency to degrade these herbicides. A positive correlation was found between its laccase activity and the corresponding herbicide degradation rate. Even more, the doubling of the enzyme activity in this phase corresponded with a doubling of the herbicide degradation rate. It is, therefore, tempting to speculate that laccase is the most dominant enzyme in the degradation of 2,4-D and 2,4,5-T under these conditions. In addition, it was shown that *Rigidoporus* sp. FMD21 partly relies on cytochromes P450-type for the breakdown of the herbicides as well. Two filamentous fungi were isolated from soil contaminated with herbicides and dioxins located at Bien Hoa airbase. They belong to genera *Fusarium* and *Verticillium* of the phylum Ascomycota as judged by their 18S rRNA gene sequences. Both isolated fungi were able to degrade the herbicides but with different rates. Their laccase activity, however, was very low and did not correlate with the rate of breakdown of the herbicides. These data indicate that the white-rot fungus most likely synthesizes laccase and cytochromes P450-type for the breakdown of the herbicides, while the types of enzyme used for the breakdown of the herbicides by the two Ascomycota remain unclear.

**Graphical abstract:**

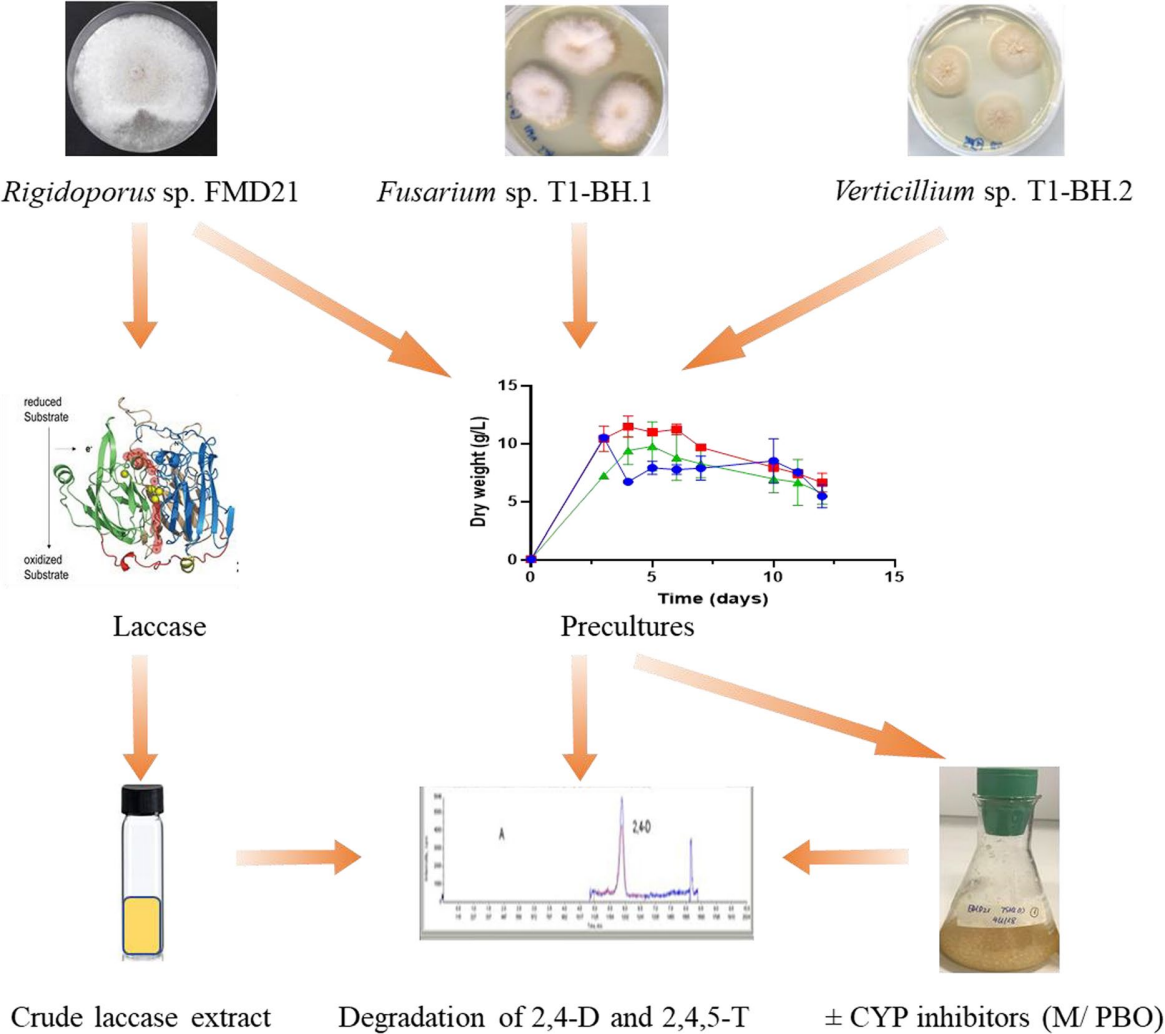

**Supplementary Information:**

The online version contains supplementary material available at 10.1007/s10532-022-09982-1.

## Introduction

The herbicides 2,4-dichlorophenoxyacetic acid (2,4-D) and 2,4,5-trichlorophenoxyacetic acid (2,4,5-T) are synthetic auxins that have been applied widely for the control of broadleaf weeds and as a plant growth-regulator in agriculture since the 1940s. They were also used as defoliating agents in the Malayan Emergency from 1948 to 1960 and during the Vietnamese war from 1961 to 1971. However, since the 1980s, 2,4,5-T is forbidden worldwide due to its toxicity in animals (Al-Fathi et al. 2019; Hayashi et al. 2016). Both herbicides are pollutants of groundwater as they are highly soluble in water on the one hand (Donald et al. 2007; Gilliom 2007) and slowly biodegraded on the other hand (Navalón et al. 2002). Therefore, bioremediation of polluted soils and groundwater by microorganisms has been of major interest in recent years because this technique is safe, relatively efficient, eco-friendly and cost-effective (Watanabe 2001). In one of these studies, an active landfill under both aerobic and anaerobic conditions was set up to detoxify soil heavily contaminated with herbicides and dioxins on-site at the former Da Nang military airbase in Central Vietnam. The results showed that the herbicides, 2,4-D and 2,4,5-T were degraded approximately 78% and 68%, respectively, during 6 months of treatment (Dang et al. 2010). Another study on a pilot plant in Da Nang that operated for 6 months showed that 2,4-D and 2,4,5-T were indeed rapidly degraded under both aerobic and anaerobic conditions (Allen 2015). We have also demonstrated in a previous study that bacterial communities isolated from soil from Bien Hoa airbase polluted with herbicides completely degraded 2,4-D and 2,4,5-T within 5 days of cultivation (Nguyen et al. 2021).

However, little is known about the biochemistry of 2,4-D and 2,4,5-T degradation by fungi although many of them can degrade these herbicides and use them as a carbon and energy source (Serbent et al. 2019). The best-known fungi that degrade 2,4-D belong to the genera *Aspergillus* (Vroumsia et al. 2005), *Penicillium* and *Trichoderma* (Bhosle and Thore 2016), *Fusarium* and *Verticillium* (Vroumsia et al. 2005) and *Umbelopsis* (Nykiel-Szymańska et al. 2018). Also, white-rot fungi such as *Phanerochaete chrysosporium*, *Pleurotus ostreatus*, *Trametes versicolor* and *Ganoderma lucidum* have a high potential for degradation of 2,4-D (Asgher et al. 2008; Coelho-Moreira et al. 2013; da Silva Coelho et al. 2010; Mendieta et al. 2018; Pointing 2001; Tortella et al. 2005). In contrast, only a few 2,4,5-T degrading fungi were reported and the best studied are *P. chrysosporium* (Yadav and Reddy 1993) and *Eupenicillium* spp. (Itoh et al. 2013).

Also, the enzymes involved in the breakdown and further metabolism of the herbicides are not well known. Some studies showed that cytochromes P450-type (CYPs) participate in the metabolism of xenobiotic compounds by some fungi such as *Aspergillus nidulans* (Ostrem Loss et al. 2019), *Coprinellus disseminatus* (Suhara et al. 2011), *Phanerochaete chrysosporium* (Coelho-Moreira et al. 2013) and *Umbelopsis isabellina* (Nykiel-Szymańska et al. 2018). CYPs are hemoproteins which belong to one of the largest protein families and are found in fungi, bacteria, insects and humans (de Montellano 2005; Nelson et al. 1996), where they, among other reactions, catalyze the degradation of aromatic compounds by various pathways (Isin and Guengerich 2007). They play a key role in the hydroxylation of pollutants in biotransformation pathways as well as in detoxification of these compounds. The degradation pathway includes multiple catalytic reactions, like hydroxylation at an unsubstituted position, hydroxylation with migration of a chloride substituent, hydroxylation with elimination of a chloride substituent and cleavage of the aromatic ring (Guengerich 1995, 2008; Hu and Bunce 1999; Kumari et al. 2021). These hydroxylation steps were shown to be essential in the 2,4-D degradation pathway with the sequential appearance of 2,4-DCP, 3,5-dichlorocatechol, 2,4-dichlorophenol (2,4-DCP), 2-chlorodienelactone and 2-chloromaleylatcetate (Smith and Beadle 2008). Other studies demonstrated the kinetically controlled attack ipso to the ether functionality as the main reaction pathway with a central role of the hydroxyl radical in the breakdown of 2,4-D and 2,4,5-T, followed by homolytic elimination of the ether side chain (Peller et al. 2003, 2004; Zona et al. 2012). More detailed studies indeed revealed an important role of a CYP-type enzyme in the breakdown of 2,4-D by *Umbelopsis isabellina*. The compound 2,4-DCP was identified as the main metabolites of fungal metabolism of 2,4-D (Coelho-Moreira et al. 2013; Nykiel-Szymańska et al. 2018). This was further reinforced by both CYP inhibitor studies and studies where the genes encoding CYPs were induced (Doddapaneni and Yadav 2004; Hiratsuka et al. 2001). To the best of our knowledge, there are no reports on 2,4,5-T degradation pathways in fungal strains.

In addition to intracellular CYPs, extracellular enzymes seem to play a role in the degradation of the herbicides as well. *P. chrysosporium* was shown to produce extracellular laccases and ligninolytic enzymes including lignin peroxidase and manganese peroxidase (Asgher et al. 2008; Jin et al. 2016; Pointing 2001; Zeng et al. 2017). Laccases are enzymes that belong to the family of multicopper oxidases containing four copper atoms in the catalytic centre which can catalyze the oxidation of various phenolic compounds, aromatic amines and a range of other aromatic compounds (Baldrian 2006; Piontek et al. 2002). According to a previous study Maruyama et al. (2006), laccase from the fungus *Trametes* sp. degraded the herbicide dymron in the presence of the mediator (ABTS) 2′-azinobis (3-ethylbenzothiazoline-6-sulfonic acid). Laccase isolated from the white-rot fungus *Rigidoporus* sp. FMD21 has been shown to degrade 2,3,7,8-tetrachlorodibenzo-*p*-dioxin (Dao et al. 2021).

The aim of this work was to determine the metabolic potential of selected fungi to degrade 2,4-D and 2,4,5-T and to reveal the potential role of CYPs and laccase involved in this process. The selected strains were *Rigidoporus* sp. FMD21 along with two newly isolated fungi from soil from Bien Hoa airbase, which is heavily contaminated with these types of herbicides. The approach was (i) to determine the degradation rate of the herbicides by these 3 fungal species using LC–MS/MS, (ii) to correlate their laccase activities to the degradation rates of 2,4-D and 2,4,5-T during their cultivation by using a specific laccase activity assay, and (iii) to determine a possible role of their CYP-type enzymes by using CYP inhibitors during degradation of these herbicides.

## Material and methods

### Fungal strains

The white-rot fungus *Rigidoporus* sp. FMD21 used in this study belongs to the microbial culture collection of the Institute of Biotechnology, Vietnam Academy of the Science and Technology, Hanoi, Vietnam. *Rigidoporus* sp. FMD21 was isolated from decayed wood in Ma Da forest, Dong Nai in the South of Vietnam with a history of exposure to herbicides during the Vietnamese war (Dao et al. 2019). This fungus was selected for its capacity to produce high amounts of laccase and its potential to degrade various xenobiotics (Dao et al. 2019).

Two filamentous fungi were isolated from soil at Bien Hoa airbase (10° 58′ 14.3″ N 106° 48′ 19.3″ E), Dong Nai Province, which is heavily contaminated with dioxins and herbicides. For that, five grams of soil were added in 45 ml of saline 0.85% and shaken for 15 min before making a series of dilutions from 10^–1^ to 10^–4^ also in saline 0.85%. One hundred microliters of each dilution were plated on the Sabouraud Dextrose Agar (SDA) plates containing antibiotics (100 µg/ml chloramphenicol and 100 µg/ml ampicillin) (Pradeep et al. 2013). The plates were incubated at 30 °C and examined daily for the growth of fungi. Each morphologically unique fungal colony was transferred to a fresh SDA plate for isolation and identification.

### Classification of the isolated fungal strains

Mycelia and fungal cells, grown in Sabouraud Dextrose Broth (SDB) medium, were harvested by centrifugation at 12,000 rpm for 10 min for DNA isolation. Isolation of DNA was carried out using the MoBio PowerSoil® DNA Isolation kit (Carlsbad, CA, USA) and following the manufacturer’s instructions. The 18S rRNA gene was amplified by polymerase chain reaction (PCR) in a Thermocycler (Biometra, Analytik Jena, Germany) using the primer pairs NS1 (5′-CCAGTAGTCATATGCTTGTC-3′) and NS2 (5′-GAATTACCGCGGCTGCTGGC-3′) (White et al. 1990). One microliter of DNA (~ 25 ng) was added to 25 μl reaction volume containing 12.5 μl of GoTaq® PCR Master Mix 2x (Promega, Netherlands), 9.5 μl nuclease-free water and 10 µM of each primer. The PCR program was carried out at 94 °C for 3 min, followed by 30 cycles of 60 s at 94 °C, 30 s at 50 °C and 60 s at 72 °C, and a final extension at 72 °C for 5 min. PCR amplicon was sequenced by Macrogen Europe BV. Partial sequences of the 18S rRNA genes from the two isolated fungal strains obtained after PCR amplification have been deposited in the GenBank database under accession numbers MN463097 (*Fusarium* sp. T1-BH.1) and MN463098 (*Verticillium* sp.T1-BH.2). They were compared against sequences of reference organisms available in the NCBI database and MEGA version 7 software was used to align and construct a phylogenetic tree by the neighbour-joining method with 1000 bootstrap replicates (Kumar et al. 2016).

### Laccase production of the white-rot fungus *Rigidoporus* sp. FMD21 and two isolated fungi

Laccase from white-rot fungus *Rigidoporus* sp. FMD21 was produced as described previously (Dao et al. 2019). The two isolated fungi were initially grown on potato dextrose agar (PDA) medium in a Petri dish within 7 days, after which 3 mycelial plugs (1 cm diameter each) from the plate were transferred to 250 ml flasks containing 100 ml of potato dextrose broth (PDB) medium. After 5 days of cultivation, inocula were transferred to 100 ml of two different liquid media including yeast extract malt extract broth (YMB) and PDSRb (potato dextrose powder soy meal rice bran) (Dao et al. 2019; Pradeep et al. 2013). All these media were adjusted to pH 6.0. These flasks were cultivated for 7 days at 30 °C and 200 rpm. The culture liquid was then centrifuged at 10,000 rpm for 10 min at 4 °C, and the supernatant was collected and used for the laccase enzyme assay. The fungal growth at different cultivation periods was determined by vacuum filtering through pre-weighed filter paper. Then, the mycelia were dried to constant weight at 80 °C in an oven and this weight was recorded. The dry weight (DW) is expressed as grams per litre of liquid medium. All experiments were performed in triplicate.

### Laccase assay

Laccase activity was determined by following the oxidation of ABTS as the substrate for laccase according to Bourbonnais et al. (1998). The enzyme activity was assayed at 30 °C using 0.5 mM ABTS in 20 mM acetate buffer pH 3 and a suitable amount of enzyme. ABTS oxidation was followed by monitoring the changes in absorbance at 420 nm (ε = 3600 M^−1^ cm^−1^) against a blank with 20 mM acetate buffer pH 3 during 2 min of incubation. All assays were performed in triplicate in flat bottom 96 well microplates and the absorbance was measured using a Multiskan™ GO UV/Vis spectrophotometer (Thermo Scientific). One unit was defined as the amount of laccase that oxidized 1 μmol of ABTS substrate per minute.

### Degradation assays of 2,4-D and 2,4,5-T

The fungi were precultivated in YMB medium or PDSRb medium for 7 days and then used as inoculum for the herbicide degradation experiment. *Rigidoporus* sp. FMD21 and the two isolated fungi were initially grown on PDA plates. Pre-inocula of the three fungal strains were prepared separately in 250 ml flasks containing 100 ml liquid culture medium of YMB (Pradeep et al. 2013) or PDSRb (Dao et al. 2019). Flasks were shaken constantly at 200 rpm for 7 days at 30 °C. Then, 5 ml of these cultures were used as inoculum (5% v/v) for a second set of 250 ml Erlenmeyer flasks containing 95 ml of basal salts medium (BS: KH_2_PO_4_ 0.5 g/l, (NH_4_)_2_SO_4_ 0.25 g/l, MgSO_4_ 0.2 g/l, CaCl_2_ 0.5 g/l and NaNO_3_ 0.4 g/l, pH 7.0) or PDSRb medium. Herbicides 2,4-D (> 95% purity) and 2,4,5-T (> 95% purity) were purchased from Sigma Aldrich (Netherlands), dissolved in acetone and then added to cultures at a final concentration of 200 mg/l and 100 mg/l, respectively. Different media such as PDSRb-PDSRb, PDSRb-BS, YMB-BS, washed YMB-BS (listed are pre-culture medium and experimental medium, separated with a dash) were used in the herbicide degradation test. The inoculum for the culture called washed YMB was cultivated on YMB for 7 days, and then centrifuged. The supernatant was removed and the pellet was washed 5 times with potassium phosphate buffer (pH 7) to remove the remaining laccase in these cultures. Two additional controls, including heat-inactivated mycelium and a non-inoculated sample, were also included. The flasks were inoculated at 30 °C in the dark for 16 days at 200 rpm on a rotary shaker. Each sample was analyzed in parallel for fungal DW, laccase activities and concentrations of the herbicides at various time points. For that, the fungal cultures were centrifuged at 12,000 rpm for 10 min at 4 °C to separate supernatant and mycelia. The supernatant was used to determine laccase activity and the herbicide concentrations, the mycelium was weighted to determine the DW. All experiments were performed in triplicate.

### Degradation of 2,4-D and 2,4,5-T by an extract of extracellular enzymes (ExE) from *Rigidoporus* sp. FMD21

Degradation of 2,4-D and 2,4,5-T by an ExE from the strain *Rigidoporus* sp. FMD21 (a total of 20 U of laccase) was carried out in 2 ml reaction vials containing 20 mM sodium acetate buffer pH 4 or phosphate buffer pH 7.0, 100 mg/l of 2,4-D or 100 mg/l of 2,4,5-T. At different time points, destructive sampling was applied for subsequent analyses. A control test contained the same amount of heated-inactivated ExE, 2,4-D and 2,4,5-T in the 20 mM sodium acetate buffer (pH 4). All experiments were carried out in triplicate. Both degradation and control tests were incubated at 30 °C for 24 h on a rotary shaker (200 rpm) in the dark.

### Effect of CYP inhibitors on herbicide degradation

Cultures, growing in 250 ml Erlenmeyer flasks containing 100 ml of YMB medium (Pradeep et al. 2013), were inoculated with 3 agar plugs (1 cm diameter) cut out from mycelia on potato dextrose agar (PDA) plates of *Rigidoporus* sp. FMD21 and the isolated fungi. The flasks were incubated at 30 °C and were shaken constantly at 200 rpm for 7 days. Piperonyl butoxide (PBO) and metyrapone were purchased from Sigma Aldrich (Netherlands) and Cayman Chemical (Netherlands), respectively, and used as CYP inhibitors for degradation assays. The homogeneous pre-culture (5% v/v) was transferred to fresh YMB medium supplemented with 200 mg/l of 2,4-D and 100 mg/l of 2,4,5-T in the presence or absence of metyrapone (2 mM) or PBO (1 mM). A control was prepared by using heat-inactivated mycelium. All flasks were incubated in the dark at 30 °C on a rotary shaker (200 rpm). Cultures were sampled over time for analysis of laccase activity, DW and concentration of herbicide by LC–MS/MS. All experiments were performed in triplicate.

### Determination of 2,4-D and 2,4,5-T concentrations by tandem liquid chromatography-mass spectrometry (LC–MS/MS)

The extraction of 2,4-D and 2,4,5-T from liquid medium was performed according to the Quick, Easy, Cheap, Effective, Rugged, and Safe (QuEChERS) extraction procedure (Rejczak and Tuzimski 2015). At different time points, aliquots of the cultures (2 ml) were transferred to 15-ml Falcon tubes. Next, 2 ml of acetonitrile with 1% formic acid was added and vortexed for 15 min. A mixture of salts (2 g of MgSO_4_, 0.5 g of NaCl, 0.5 g of C_6_H_5_NaO_7_·2H_2_O, and 0.25 g of C_6_H_6_Na_2_O_7_·1.5 H_2_O) was added to the homogenate and mixed for 5 min, followed by centrifugation for 5 min at 12,000×*g*. Then, 2 ml of the supernatant was collected for the LC–MS/MS analysis. The degradation rate was determined by measuring the amount of residual 2,4-D and 2,4,5-T in the culture medium in time by LC–MS/MS using an Elute ultra-high-pressure liquid chromatography (UHPLC) system coupled to an EVOQ triple quadrupole mass selective detector (TQ-MS; both Bruker, Bremen Germany). The target compounds were separated on a 50 × 2 mm, 3 µm Luna C18 column (Phenomenex, Utrecht, The Netherlands) at a temperature of 40 °C, applying a gradient of 0.1% formic acid and methanol (both Biosolve, Valkenswaard, The Netherlands). The gradient started with 5% methanol, increased in 2 min to 30% followed by an elevation to 100% in 4 min with a constant flow of 400 µl/min. The TQ-MS was set at electrospray ionization in a negative ion mode. The transitions for 2,4-D were 221 > 163 and 219 > 161 (quantifier/qualifier). For 2,4,5-T the transitions were 253 > 195 and 255 > 197 (quantifier/qualifier). All the transitions were scanned with a rate of 300 ms. Before analysis, samples were diluted in deionized water and stored at 4 °C until injection. Data acquisition and analyses were performed with MS Data review software. Data analyses of the biodegradation tests were performed using Prism version 8.2.1.

## Results

### Fungal identification

Two fungi were isolated from soil exposed to dioxins and herbicides at Bien Hoa airbase, Vietnam (Fig. S1 in Supplementary file). The partial 18S rRNA sequences of the 2 strains were more than 99% identical to those of the closest homologues in the NCBI database, *Fusarium oxysporum* (accession number CP053261.1) and *Verticillium dahliae* (accession number CP010980.1), successively (Fig. S2 in Supplementary file). Therefore, the new isolates were tentatively termed *Fusarium* sp. T1-BH.1 and *Verticillium* sp.T1-BH.2. Both fungi belong to the phylum of Ascomycota.

### Growth curves of the three fungal strains and their laccase production

**Fig. 1:**
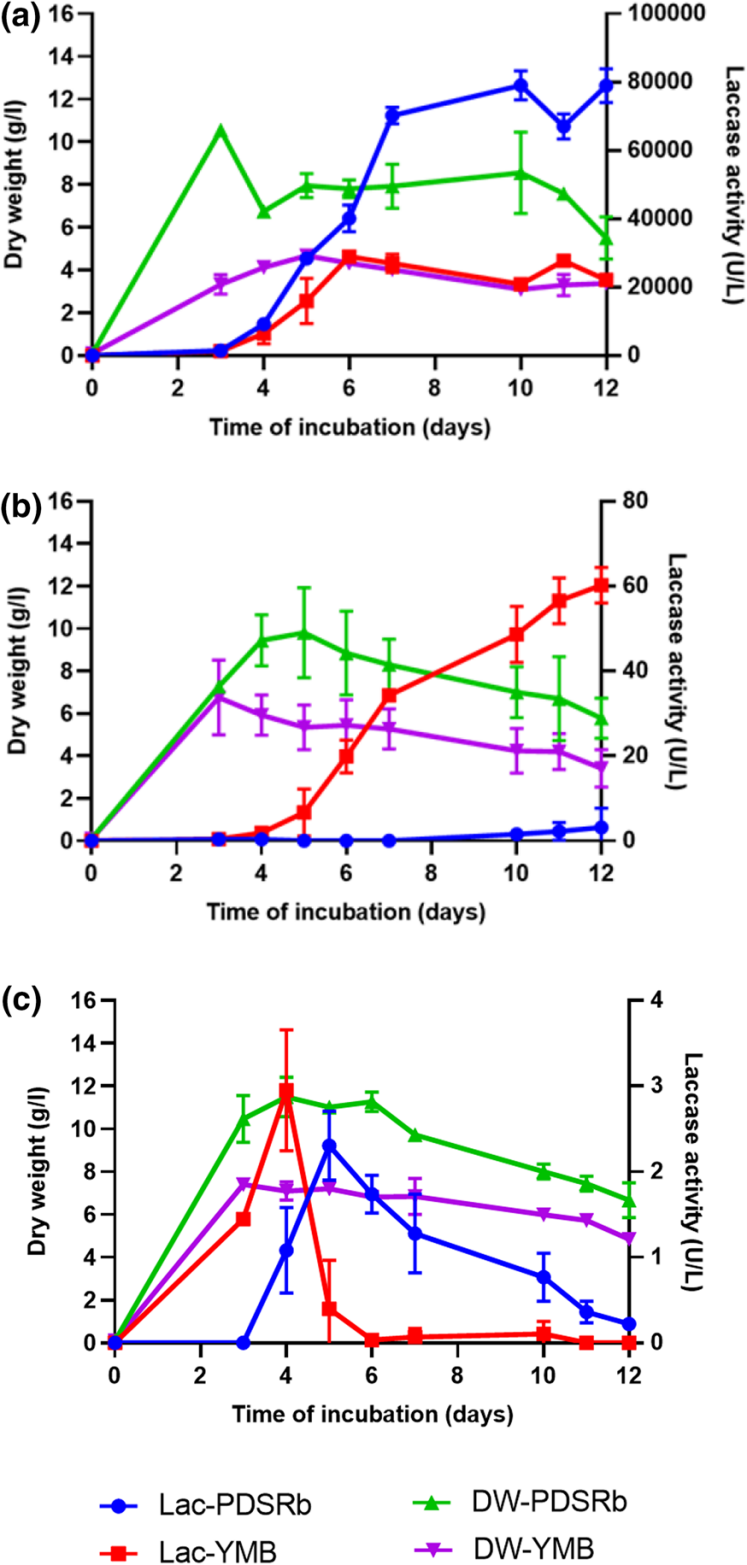
Growth curves and laccase production of strain *Rigidoporus* sp. FMD21 (**a**), strain *Fusarium* sp. T1-BH.1 (**b**) and strain *Verticillium* sp. T1-BH.2 (**c**) grown on PDSRb and YMB media. The error bar at each data point represents the standard error of three independent experiments

The growth curves of the three fungal strains are shown in Fig. 1 along with their laccase activities. Both growth rate and yield of *Rigidoporus* sp. FMD21 were higher on PDSRb medium than on YMB medium. This also holds for the laccase activity, which reaches around 80,000 U/l in PDSRb medium as opposed to around 28,000 U/l in YMB medium. This result agrees with data from Dao et al. (2019), who showed earlier that strain *Rigidoporus* sp. FMD21 secreted the highest laccase activity in PDSRb medium. Remarkably, the optimal laccase activity is reached after around 6 to 8 days in both media, whereas the maximum growth yield is apparent already after 3 to 4 days. *Fusarium* sp. T1-BH.1 and *Verticillium* sp. T1-BH.2 grew with quite similar growth characteristics as compared to *Rigidoporus* sp. FMD21 concerning specific growth rate and yield and also medium dependent with PDSRb medium showing the best results.

These two strains, however, displayed much lower laccase activities. *Verticillium* sp. T1-BH.2 peaks at around 3 U/l after 4 days and 2 U/l after 5 days of incubation in YMB and PDSRb media, respectively. Whereas it is slightly better in *Fusarium* sp. T1-BH.1, which produces around 60 U/l in YMB medium and around 3 U/l in PDSRb medium after 12 days of incubation. Altogether, they produce just a fraction of what is produced by *Rigidoporus* sp. FMD21.

### Degradation of 2,4-D and 2,4,5-T on different media by the three fungal strains

**Fig. 2:**
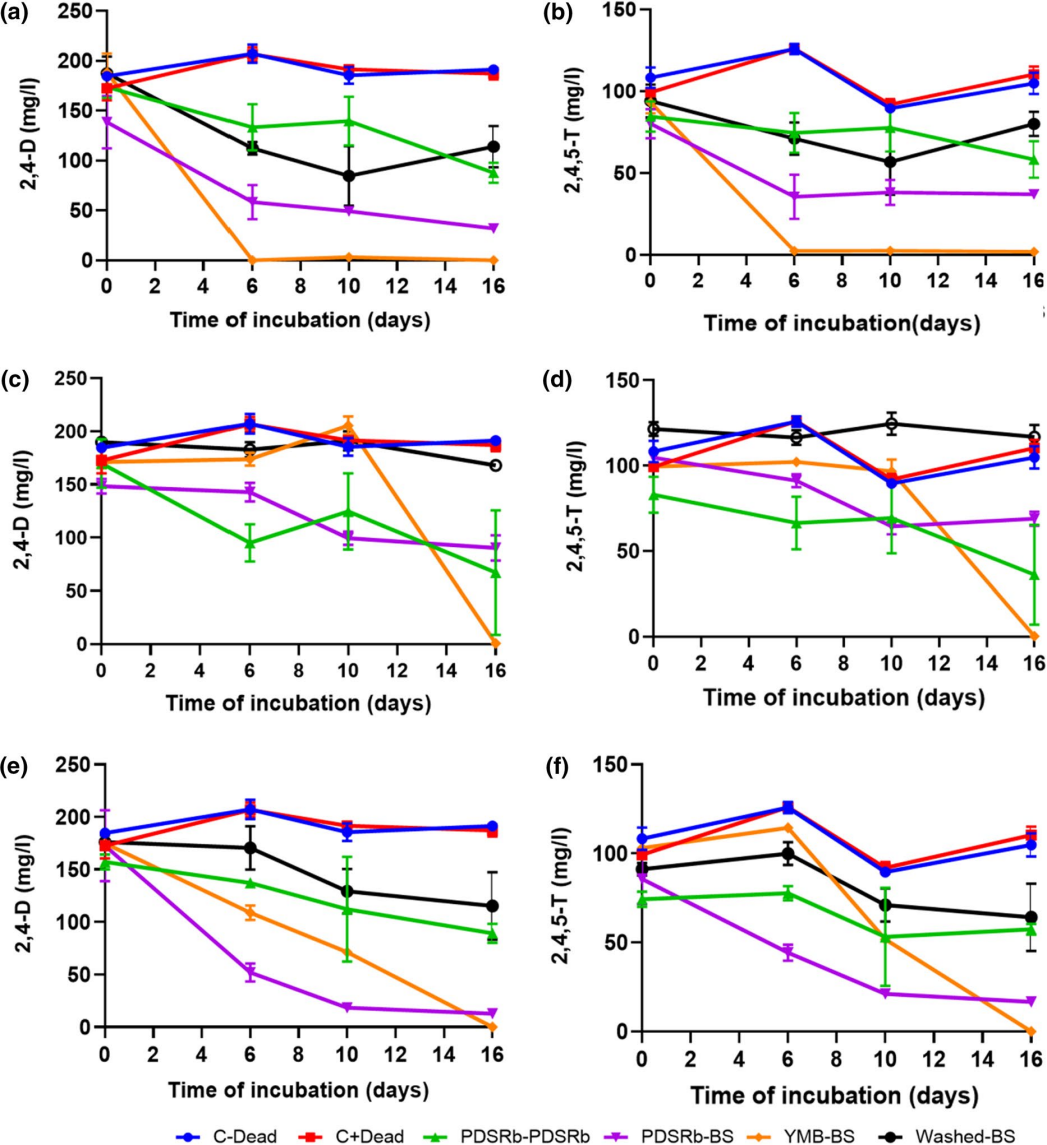
Concentrations of 2,4-D and 2,4,5-T of *Rigidoporus* sp. FMD21 (**a**, **b**) *Fusarium* sp. T1-BH.1 (**c**, **d**) and *Verticillium* sp. T1-BH.2 (**e**, **f**) growing on different media. The error bars at the data points are the standard errors of three independent experiments. Control cultures contain MS medium with heat-denatured dead cells (C + Dead) or without dead cells (C-Dead)

The three fungal strains were cultivated in BS medium containing 2,4-D and 2,4,5-T as sources of carbon and energy. Their DW, laccase activity and the degradation of 2,4-D and 2,4,5-T by these fungi were determined over time and the results are presented in Fig. S3 and Fig. 2*.* The DW of all three fungi growing in the PDSRb-PDSRb medium increased and reached a maximum after 6 days of cultivation. All fungal strains showed poor growth in BS medium with a slight increase in DW after 6 days of cultivation, which then decreased upon further cultivation (Fig. S3b, c and d in Supplementary file).

Laccase was hardly produced by *Fusarium* sp. T1-BH.1 and *Verticillium* sp. T1-BH.2 regardless of the medium (data not shown). The laccase activities from *Rigidoporus* sp. FMD21 at the start of these cultures were obtained from pre-cultures on PDSRb or YMB media, and were in between 3200 and 3800 U/l. These values reduced over time in all media (Fig. S3 in Supplementary file). The control was a culture inoculated with a washed pre-culture that lacked laccase activity.

In general, the degradation kinetics of both 2,4-D and 2,4,5-T were quite similar for each fungus (Fig. 2). Control cultures did not show the removal of 2,4-D and 2,4,5-T*. Rigidoporus* sp. FMD21 growing in YMB-BS and PDSRb-BS medium degraded these herbicides with a half-time of around 3 and 6 days of cultivation, respectively. *Rigidoporus* sp. FMD21 fully degraded both herbicides in YMB-BS medium after 6 days of cultivation (Fig. 2a, b). The laccase activity was around 1100 U/l and the biomass increased to 0.13 g/l in YMB-BS medium after 6 days of incubation (Fig. S3a and b). Similarly, *Fusarium* sp. T1-BH.1 completely degraded the herbicides only in YMB-BS medium with an increase in biomass of 1.29 g/l after 16 days of cultivation and hardly in the other media. For that, it needed a lag-phase of around 10 days, after which both herbicides were entirely removed within 16 days of cultivation (Fig. 2c, d). *Verticillium* sp.T1-BH.2 growing in PDSRb-BS and YMB-BS media degraded the herbicides with a half-time of around 4 and 9 days of cultivation with the increase of biomass of around 0.1 g/l and 0.23 g/l, respectively (Fig. 2e, f). The half-time of degradation was more than 16 days in the other cultures. *Verticillium* sp. T1-BH.2 completely degraded both herbicides in YMB-BS medium after 16 days (Fig. 2e, f).

**Fig. 3:**
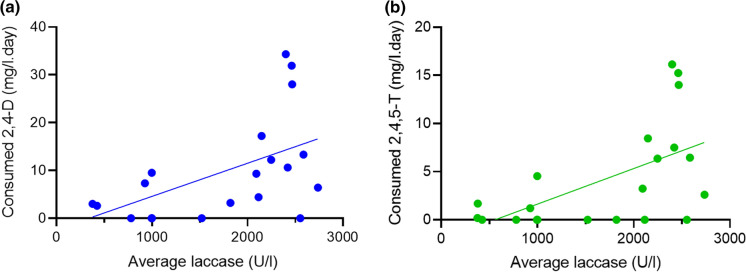
Correlations between the daily consumption of the herbicides 2,4-D (**a**) and 2,4,5-T (**b**) and the average laccase activities of *Rigidoporus* sp. FMD21 with p-values of the Spearman rank correlations < 0.05

From the above experiments, we calculated the average laccase activity from *Rigidoporus* sp. FMD21 and correlated these to the concentrations of 2,4-D and 2,4,5-T that were consumed per day. We then determined a Spearman’s correlation coefficient and p-value for these correlations (Tables S1 and S2). The correlation analyses were not done for the Ascomycota since these two fungi hardly produced laccases. But we did notice a positive correlation between laccase activity of *Rigidoporus* sp. FMD21 (Fig. 3a and b). FMD21 and its herbicide consumption rate as judged by the Spearman’ rank correlation coefficient. Even more importantly, the plot shows clearly that the rate of 2,4-D degradation doubles at a doubling of the laccase activity.

### Degradation of 2,4-D and 2,4,5-T using an extract of extracellular enzymes (ExE)

**Fig. 4:**
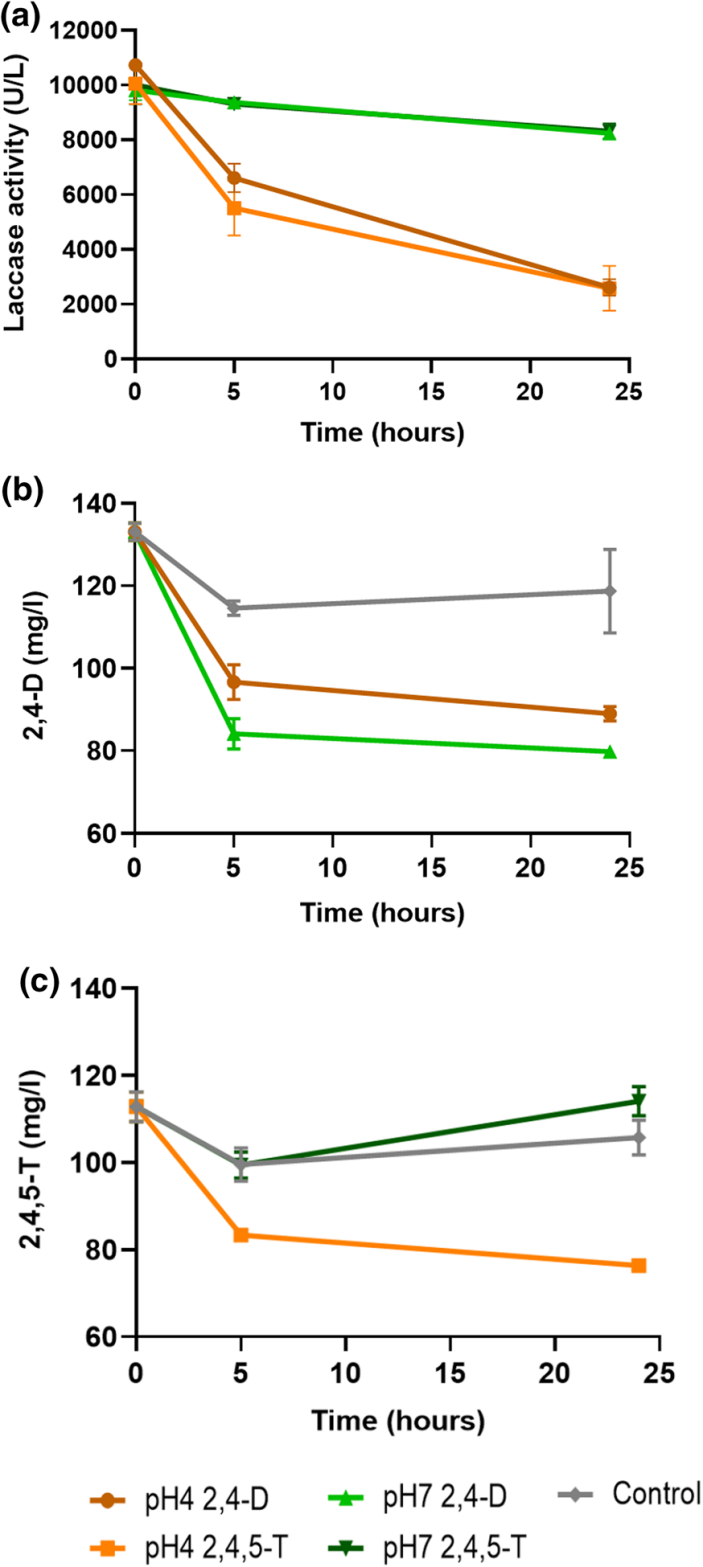
Laccase activity (**a**) and degradation of 2,4-D (**b**) and 2,4,5-T (**c**) by an ExE from *Rigidoporus* sp. FMD21 in buffers at pH 4 and pH 7. The control contains a mixture of 2,4-D, 2,4,5-T and heat-denatured ExE. The error bar at each data point represents the standard error of three independent experiments. The data are obtained after the analyses of extracts from sacrificed cultures

We then studied the degradation of 2,4-D and 2,4,5-T by an ExE from strain *Rigidoporus* sp. FMD21 in different buffers (Fig. 4). The laccase activity was relatively stable in phosphate buffer pH 7 during 24 h of the experiment, but it decreased in time in the sodium acetate buffer pH 4 (Fig. 4a) with a half-time of around 5 h.

Degradation of 2,4-D in buffers pH 4 and pH 7 was quite comparable, though it went slightly faster at pH 7. After 5 h of incubation, however, the 2,4-D concentration remained almost constant at both pH values (Fig. 4b). The degradation rate of 2,4,5-T was the highest at pH 4, but also we observed that the degradation rate slowed down severely after 5 h (Fig. 4c). Surprisingly, 2,4,5-T was not removed at pH 7. These results clearly indicate pH effects on the stability of laccase and, consequently, the degradation kinetics in the biochemical assay.

### Degradation of 2,4-D and 2,4,5-T in the presence and absence of CYP inhibitors

**Fig. 5:**
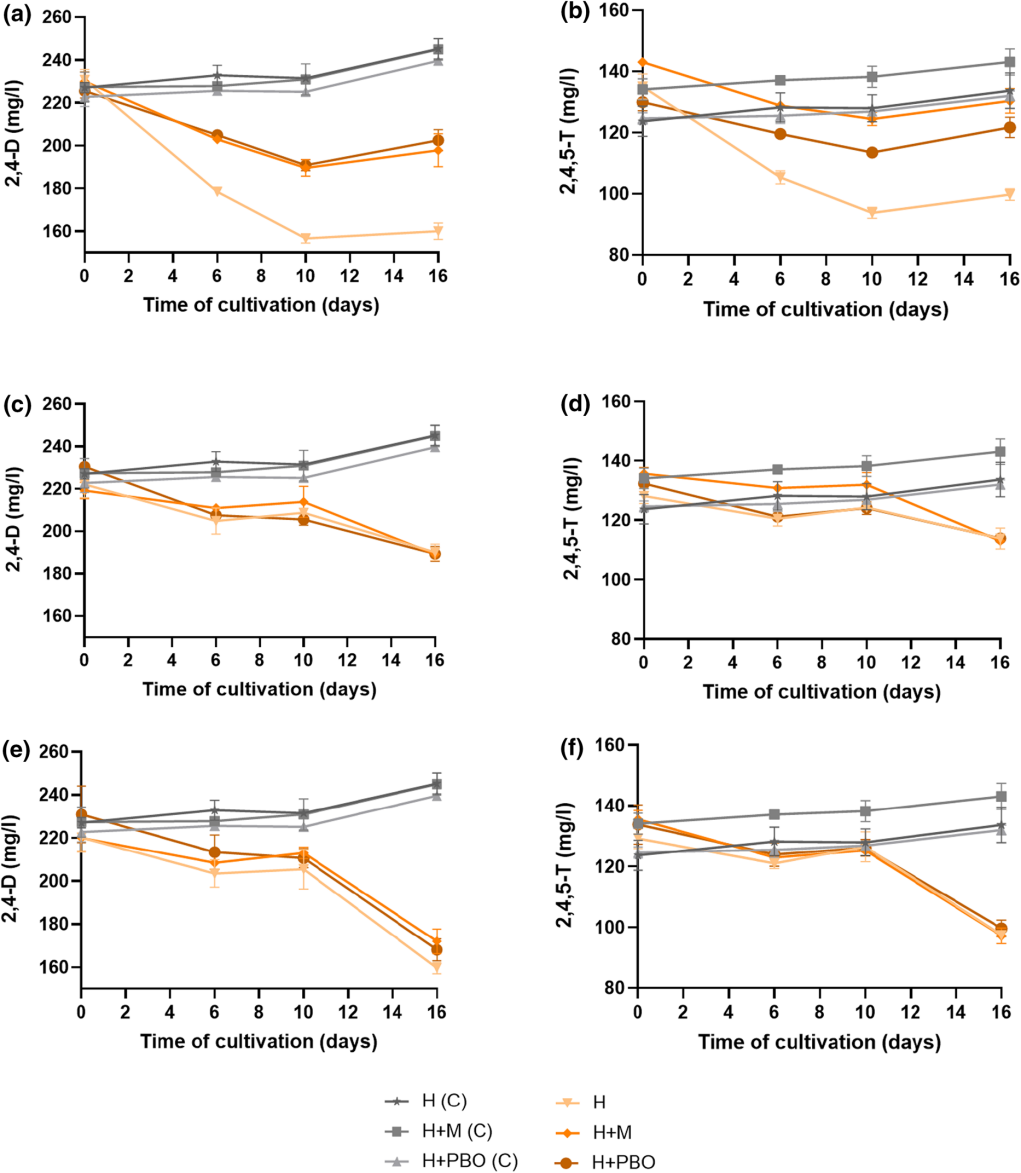
Degradation of 2,4-D and 2,4,5-T in cultures of *Rigidoporus* sp. FMD21 (**a**, **b**), *Fusarium* sp. T1-BH.1 (**c**, **d**) and *Verticillium* sp. T1-BH.2 (**e**, **f**) in the presence or absence of the CYP inhibitors metyrapone (M) or piperonyl butoxide (PBO). H: Herbicides 2,4-D and 2,4,5-T. Control incubations (C) consisted of heat denatured fungal cells and both herbicides with and without the inhibitors metyrapone (M) or piperonyl butoxide (PBO)

In this experiment, we aimed at getting a better understanding of the involvement of a CYP-type enzyme in herbicide degradation. We set up a growth experiment where the fungi were cultivated in YMB medium supplemented with 2,4-D and 2,4,5-T in the presence and absence of CYP inhibitors such as metyrapone and PBO. The results of their DW and herbicide degradation are shown in Fig. S5 and Fig. 5. The DW of *Rigidoporus* sp. FMD21, *Fusarium* sp. T1-BH.1, and *Verticillium* sp. T1-BH.2 reached a maximum after 4, 6, and 2 days, respectively, regardless of the presence or absence of the CYP inhibitors (Fig. S5a, b and c in Supplementary file). It shows that the CYP inhibitors did not affect the growth of these fungi. Upon longer cultivation, the DW of all fungi slightly decreased.

There was 20% less herbicide removal by strain *Rigidoporus* sp. FMD21 in the culture with the CYP inhibitors metyrapone or PBO as compared to that in the inhibitor-free culture after 10 days of cultivation (Fig. 5a and b). This result suggested the involvement of an intracellular CYP in 2,4-D and 2,4,5-T degradation by strain *Rigidoporus* sp. FMD21. Nevertheless, the influence of the CYP inhibitors on the ability of herbicide degradation was not observed in the other two fungal strains, *Fusarium* sp. T1-BH.1 and *Verticillium* sp. T1-BH.2. The same herbicide degradation was observed by the latter two strains in the medium with or without the CYP inhibitors after 16 days of cultivation (Fig. 5c–f). The results also show that the degradation of 2,4,5-T is slower than that of 2,4-D, which agrees with the fac it has three chlorinated substitutions rather than in 2,4-D.

## Discussion

In this study, we confirm that the fungal strains *Rigidoporus* sp. FMD21 along with two newly isolated Ascomycota from contaminated soil from Bien Hoa airbase, *Fusarium* sp. T1-BH.1 and *Verticillium* sp. T1-BH.2, were all able to degrade 2,4-D and 2,4,5-T although at different rates and with different key enzymes for degradation of these herbicides. *Rigidoporus* sp. FMD21 most likely recruits laccases and CYP-type enzymes as judged by the positive correlation between its laccase activities and the herbicides degradation rates, and by using CYP inhibitors. *Fusarium* sp. T1-BH.1 and *Verticillium* sp. T1-BH.2 use different enzymes as we did not find such correlations for these two types of fungi, nor did we notice a possible role of their CYP-type enzymes by using CYP inhibitors during degradation of these herbicides.

To the best of our knowledge, no studies on 2,4-D and 2,4,5-T degradation by species of the genus *Rigidoporus* have been reported earlier. White-rot fungi are well known for their ability to produce high amounts of laccase as judged by studies on *Trametes* sp. (Tong et al. 2007), *Pleurotus ostreatus* (El-Batal et al. 2015), *Lenzites elegans* (Pandey et al. 2018), *Cerrena unicolor* (Zhang et al. 2018) and *Rigidoporus* sp. (Dao et al. 2019). We found a positive correlation between the laccase activity and the herbicide removal rate of *Rigidoporus* sp. FMD21, indicating that laccase is responsible for the degradation of 2,4-D and 2,4,5-T in this fungus as well. Even more, a twofold in laccase activity resulted in a doubling of the 2,4-D degradation rate showing that the rate of degradation is proportional to the enzyme activity. This conclusion is further corroborated by the demonstration that an ExE of *Rigidoporus* sp. FMD21 with high laccase activity degraded both 2,4-D and 2,4,5-T. These results concur with many other studies that demonstrated that laccase is involved in the breakdown of pesticides, herbicides and other recalcitrant pollutants (Jin et al. 2016; Pizzul et al. 2009; Zeng et al. 2017). The enzyme from *Trametes versicolor*, for example, degraded herbicides and pesticides such as chlorpyrifos, chlorothalonil, pyrimethanil, atrazine and isoproturon (Jin et al. 2016). Additionally, laccase-mediator systems can also effectively assist in these kinds of degradation processes (Zeng et al. 2017). However, no studies about the pathways of 2,4-D and 2,4,5-T degradation by laccase have been documented.

Next to laccases, CYP-type enzymes appear to be also involved in the degradation of the herbicides by *Rigidoporus* sp. FMD21 as judged by the fact that CYP inhibitors slowed down the breakdown of phenoxy herbicides. This result agrees with studies on the degradation of recalcitrant pollutants where a role of a CYP-type enzyme in the degradation of phenoxy herbicides was demonstrated (Lah et al. 2011; Makovec and Breskvar 2000; Wen-Sheng et al. 2006). That enzyme apparently catalyses the transformation of 2,4-D into 2,4-DCP (Nykiel-Szymańska et al. 2018). Nakagawa et al. (2006) proved that an endemic soil fungus *Mortierella* spp. strain degraded 2,4-DCP to form four metabolites including 3,5-dichlorocatechol (3,5-DCC), 3,5-dichloro-guaiacol (3,5-DCG) and 4,6-dichloroguanacol (4,6-DCG) and chorohydroquinone (CHQ). In the first proposed pathway, 2,4-DCP was oxidized at the ortho-position to form 3,5-DCC and then the two hydroxyl groups of 3,5-DCC were methylated to form 3,5-DCG and 4,6-DCG. In the second proposed pathway, two chloride ions of 2,4-DCP were eliminated to form CHQ and hydroquinone (HQ). Another study reported that the intermediates of the proposed pathway of 2,4-D degradation by the fungus *A. niger* were 2,4-DCP and 3,5-DCC (Shailubhai et al. 1983).

Studies on fungi that degrade phenoxy herbicides have been limited primarily to species of the genera *Fusarium* and *Verticillium*. Some of these were isolated from polluted soil and able to degrade these types of herbicides (Arfarita et al. 2014; Bordjiba et al. 2001; Eman et al. 2013). These properties are shared with *Fusarium* sp. T1-BH.1 that we isolated in this study. But not all *Fusarium* species seem to be able to do that, since a fungal strain belonging to the genus *Fusarium*, which was isolated from Vietnamese soil previously exposed to herbicides, could not degrade 2,4-D or 2,4,5-T (Itoh et al. 2013). Ascomycota have been studied for their potential to degrade aromatic substances such as polycyclic aromatic hydrocarbons (PAHs), chlorinated hydrocarbons, and diverse xenobiotics (Aranda 2016; Bovio et al. 2017; Marco-Urrea et al. 2015). They are characterized by the involvement of an intracellular enzymatic system mediated by CYP-type enzymes, and some species are also able to secrete lignin modifying enzymes (LMEs) such as manganese peroxidase, lignin peroxidase and laccase. However, the enzymes responsible for the degradation of these herbicides by the Ascomycota that we isolated remain to be identified as they hardly showed laccase activity, while CYP inhibitors did not affect the degradation capability of the herbicides either. It has been demonstrated that different fungi were able to use 2,4-D as the carbon and energy sources using alternative pathways for 2,4-D transformation without the participation of a CYP. It has been reported that *Aspergillus penicilloides* and *Mortierella isabellina* converted 2,4-D also into 2,4-DCP just like in the CYP-catalysed reaction, but the enzyme responsible for this remains to be identified (Vroumsia et al. 2005). Additional studies showed that strain *A.niger* hydroxylated the phenolic ring of 2,4-D to form the major metabolite 2,4-dichloro-5-hydroxyphenoxyacetic acid and the minor metabolite 2,5-dichloro-4-hydroxyphenoxyacetic acid (Faulkner and Woodcock 1964, 1965), but also here the key-enzymes were not found. Some hardly explored enzymes such as unspecific peroxygenases (UPOs, EC 1.11.2.1) play a main role in hydroxylation. UPOs are heme-thiolate enzymes that bring together the catalytic properties of classical peroxidases and P450-type monoxygenases (Hofrichter et al. 2020). UPOs are widely distributed amongst the Ascomycota, which are known to catalyze a broad range of oxidative transformations on aromatic compounds and drug metabolites by using hydrogen peroxide as the electron acceptor (Hofrichter et al. 2015; Poraj-Kobielska et al. 2011). To the best of our knowledge, no study on the pathway of 2,4,5-T degradation by Ascomycota has been reported thus far.

The three types of fungi that we monitored for herbicide degradation were pre-cultured in YMB or PDSRb media, which contains glucose as an additional carbon and Gibbs energy source. Growth in different media resulted in different rates of herbicide degradation. This result indicates that the composition of the culture medium affects the production and/or activity of enzymes that are involved in herbicide degradation. Indeed it has been shown that the metabolic environment of fungi can alter their gene expression levels in defined cultures (Kocarek et al. 1993). They were unable to grow on the herbicides alone as sole sources of carbon and energy. The three fungal strains had maximum growth rates on PDSRb-PDSRb medium, but their rates of herbicide degradation in this medium slowed down as compared to that in PDSRb-BS or YMB-BS medium. This phenomenon may well be explained by assuming that fungi can selectively utilize a more easily metabolizable carbon and Gibbs energy source as compared to the herbicides when multiple carbon sources are available in the culture medium. Indeed, addition of extra, more easily degradable carbon and energy sources to culture media, may slow down the degradation of pollutants as reported in many studies (Barriuso et al. 1997; Khleifat et al. 2015; Lee et al. 2020; Teng et al. 2010). We, therefore, hypothesize that the presence of these auxiliary sources supported primary metabolism for fungal growth, during which catabolic enzymes involved in secondary metabolism catalyze xenobiotic degradation. Other studies also demonstrated that such type of co-metabolism played a major role in the microbial biodegradation of xenobiotics (Carles et al. 2018; Huang et al. 2018; Zhao et al. 2020). Wang et al. showed that the degradation rate of the herbicide diuron by strain *Neurospora intermedia* DP8-1 went much faster in the presence of glucose than without it (Wang et al. 2017).

## Conclusion

Taken all our results together, we have demonstrated that laccase is important for the degradation of 2,4-D and 2,4,5-T by *Rigidoporus* sp. FMD21. In addition, we suggest that CYPs are involved in the pathway of 2,4-D and 2,4,5-T transformation by *Rigidoporus* sp. FMD21 as well. Though the Ascomycota *Fusarium* sp. T1-BH.1 and *Verticillium* sp. T1-BH.2 have the potential to degrade 2,4-D and 2,4,5-T, it is clear that they do so by an enzymatic pathway that does not include laccase. Future studies will address which enzymes are responsible for the degradation of the herbicides by these strains.

## Supplementary Information

Below is the link to the electronic supplementary material.

Supplementary file1 (DOCX 725 KB)

## Data Availability

The authors declare that all the data are available and original.
