# Supplementary material for: Degradation of 2,4-dichlorophenoxyacetic acid (2,4-D) and 2,4,5-trichlorophenoxyacetic acid (2,4,5-T) by fungi originating from Vietnam
Source: Biodegradation. 2022 May 2;33(3):301–16. doi: 10.1007/s10532-022-09982-1 (PMC9106640; doi:10.1007/s10532-022-09982-1)
Supplement: Supplementary file 1 — Supplementary file1 (DOCX 725 KB) [file 10532_2022_9982_MOESM1_ESM.docx]

**Supplementary material**


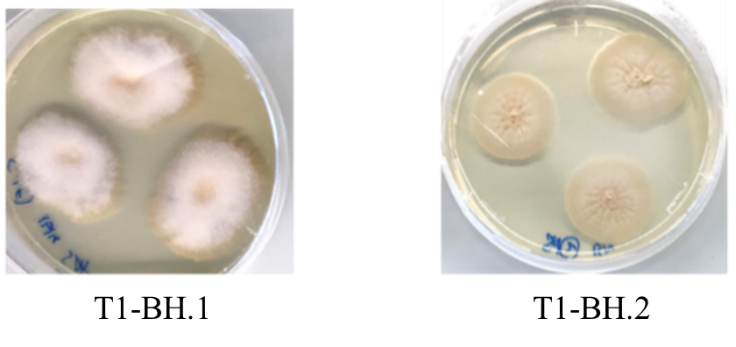


**Figure S1.** Morphology of *Fusarium* sp. T1-BH.1 (left) and *Verticillium* sp. T1-BH.2 (right) on SDA plates.


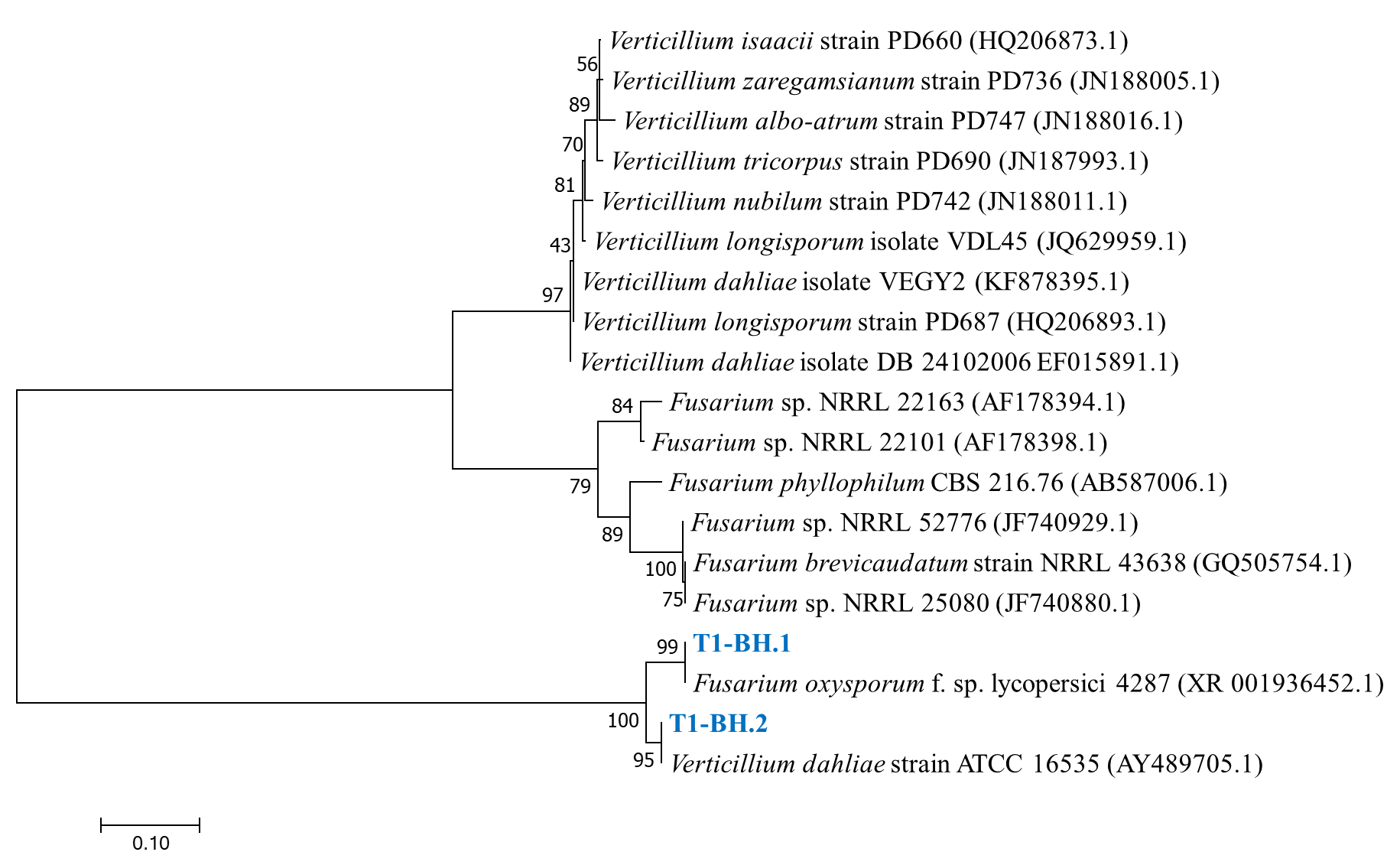


**Figure S2.** A neighbor-joining phylogenetic tree of 2 isolated fungi *Fusarium* sp. T1-BH.1 and *Verticillium* sp. T1-BH.2 compared to other fungi representative for the different phyla.


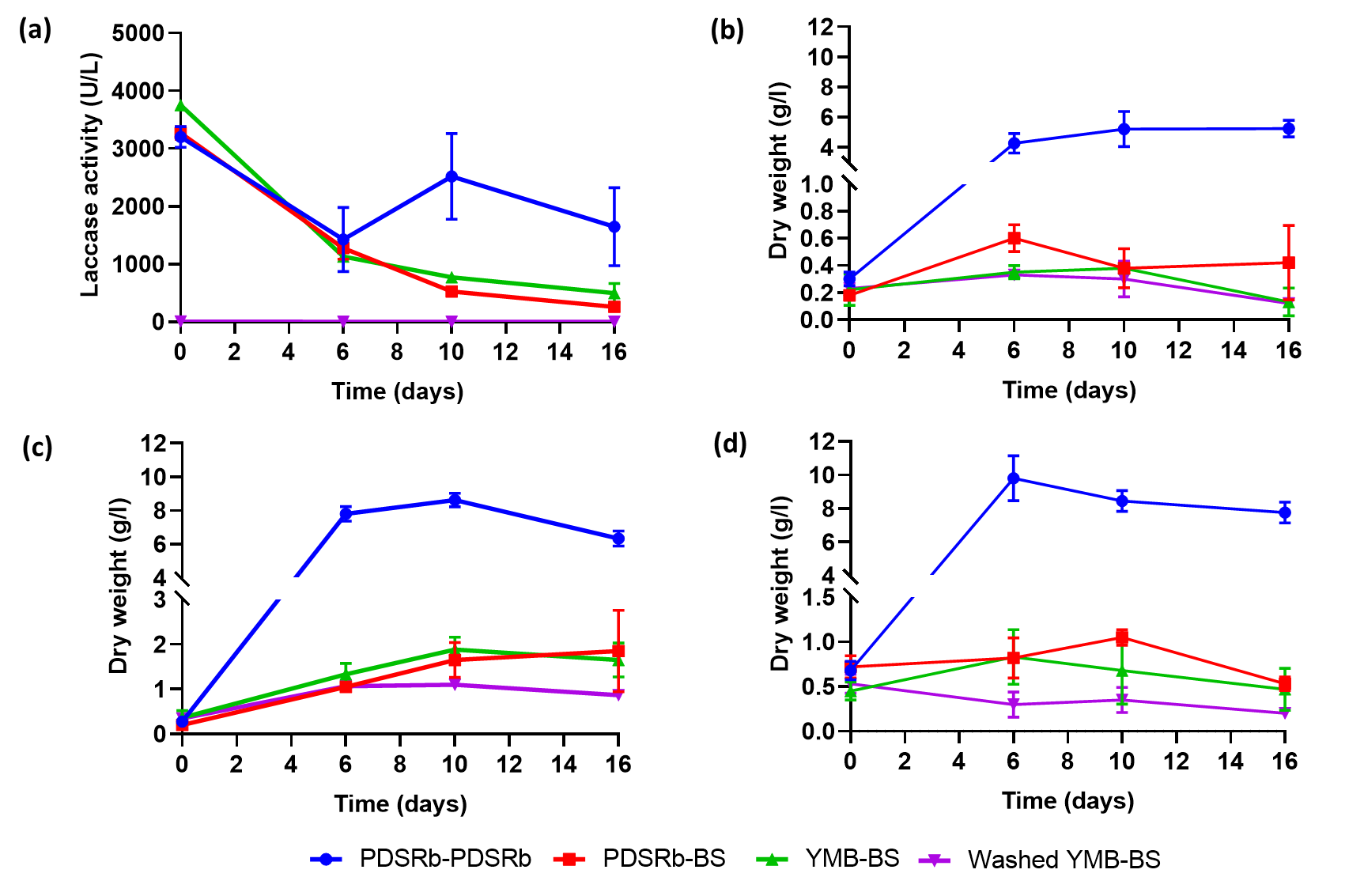


**Figure S3.** Laccase production of strain *Rigidoporus sp.* FMD21(a) and DW of strain *Rigidoporus* sp. FMD21 (b), strain *Fusarium* sp. T1-BH.1 (c) and strain *Verticillium* sp. T1-BH.2 (d) growing on different media in the presence of 2,4-D and 2,4,5-T. The error bar at each data point represents the standard error of three independent experiments. In each case, 5% (v/v) of inoculum from a pre-culture (medium type left of the dash) was transferred to fresh medium (medium type right of the dash) in the presence of 2,4-D and 2,4,5-T.


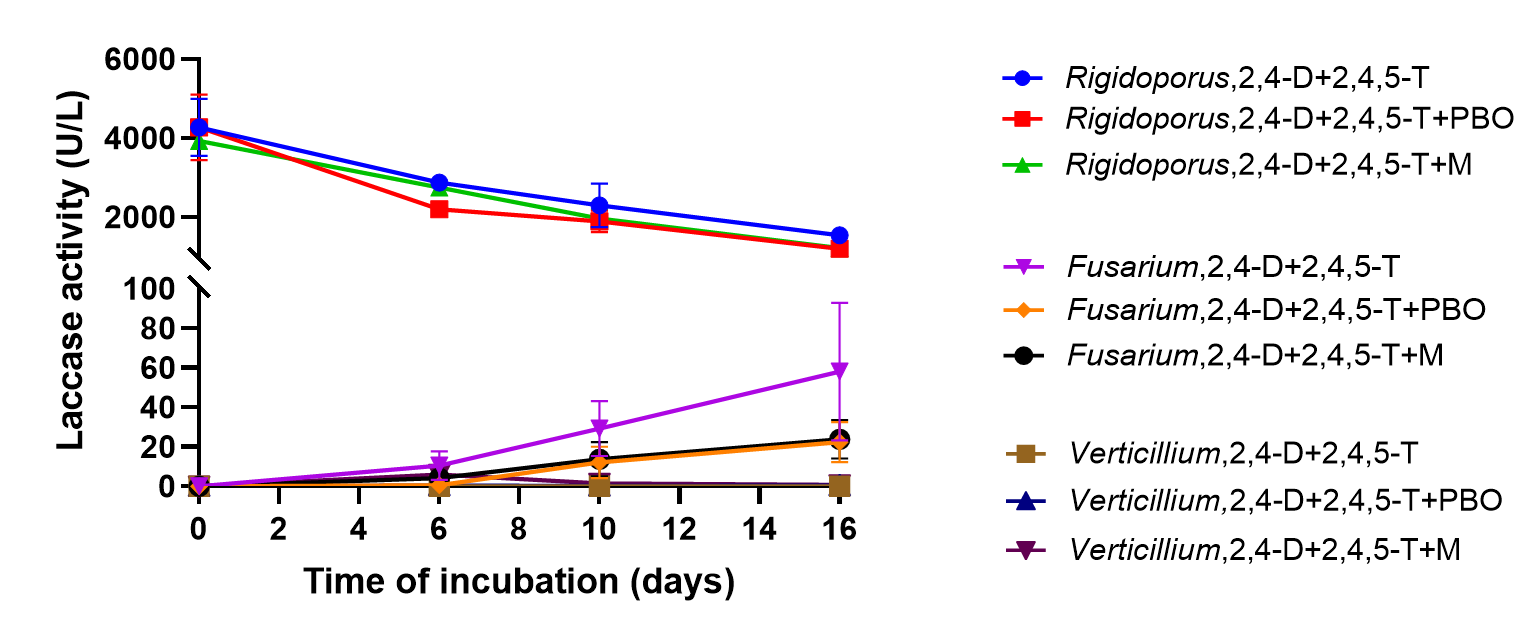


**Figure S4.** Laccase activities of *Rigidoporus* sp. FMD21, *Fusarium* sp. T1-BH.1 and *Verticillium* sp. T1-BH.2 on YMB with 2,4-D and 2,4,5-T in the presence or absence of the CYP inhibitors metyrapone (M) or piperonyl butoxide (PBO) during the enrichment.


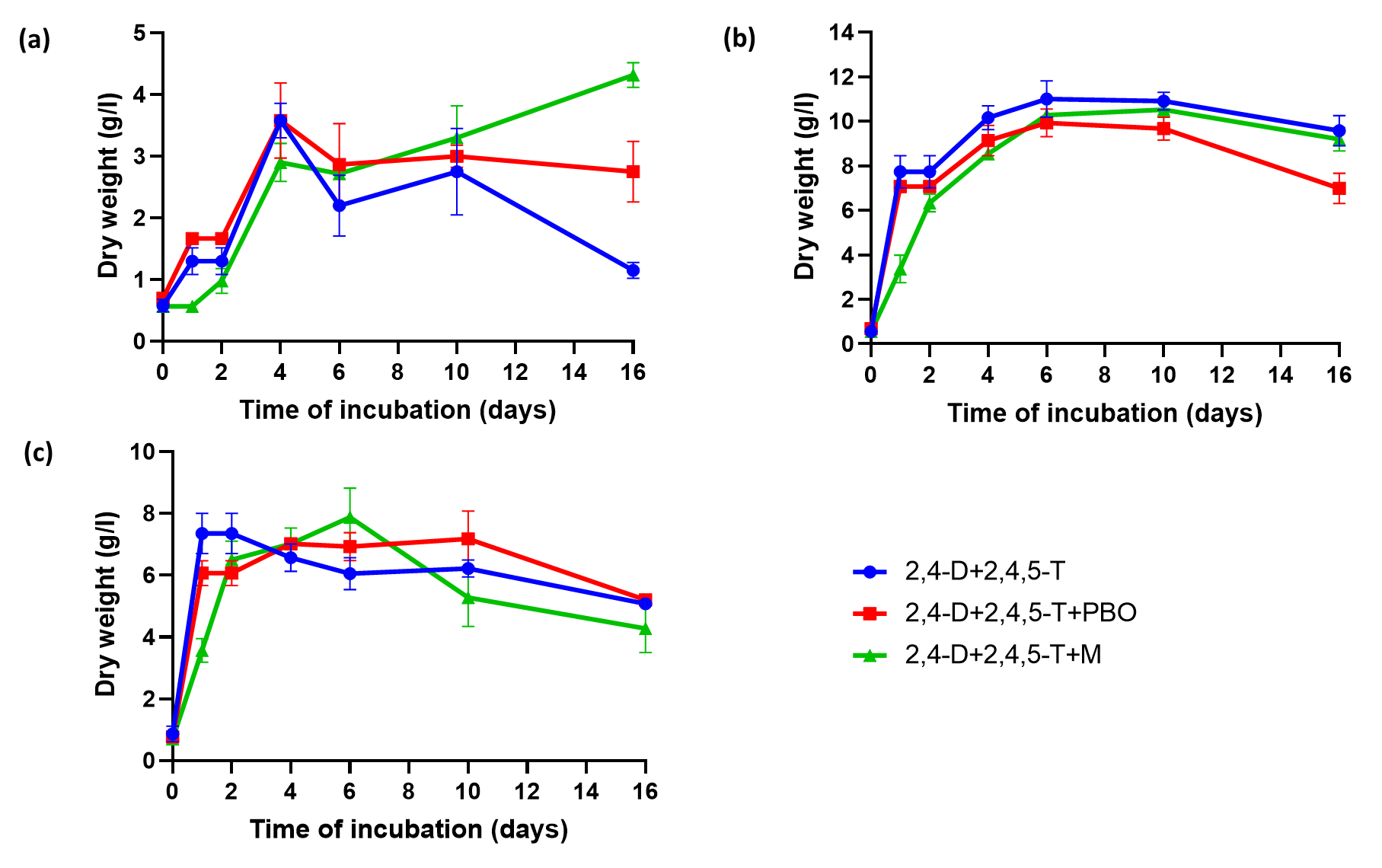


**Figure S5.** Growth of *Rigidoporus* sp. FMD21 (a), *Fusarium* sp. T1-BH.1 (b) and *Verticillium* sp. T1-BH.2 (c) on YMB medium added with 2,4-D and 2,4,5-T in the presence or absence of the CYP inhibitors metyrapone (M) and piperonyl butoxide (PBO). Data points are averages of a triplicate with the error bar representing the standard error.

**Tables**

**Table S1.** Spearman's rank correlation coefficient of average laccase activities and consumed 2,4-D (a) and statistical analysis (b)

**(a)**

| Consumed 2,4-D (mg/l.day) | Consumed 2,4-D (Rank) | Average laccase  (U/L) | Average laccase (Rank) |
| --- | --- | --- | --- |
|  |  |  |  |
| 6.4 | 11 | 2737 | 1 |
| 4.4 | 12 | 2115 | 10 |
| 9.3 | 9 | 2092 | 11 |
| 0.0 | 23 | 2552 | 3 |
| 0.0 | 23 | 995 | 17 |
| 0.0 | 23 | 1520 | 13 |
| 13.3 | 5 | 2585 | 2 |
| 9.5 | 8 | 998 | 16 |
| 3.2 | 13 | 1821 | 12 |
| 17.2 | 4 | 2147 | 9 |
| 10.6 | 7 | 2420 | 6 |
| 12.2 | 6 | 2247 | 8 |
| 0.0 | 23 | 780 | 21 |
| 0.0 | 23 | 999 | 15 |
| 7.3 | 10 | 925 | 19 |
| 3.0 | 15 | 377 | 27 |
| 2.6 | 16 | 424 | 25 |
| 3.1 | 14 | 379 | 26 |
| 34.3 | 1 | 2400 | 7 |
| 28.0 | 3 | 2467 | 4 |
| 31.9 | 2 | 2462 | 5 |
| Spearman correlation | 0.542495248 | | |

**(b)**

| Parameters | Consumed [2,4-D] |
| --- | --- |
| r | 0.542495248 |
| p | 0 |
| n | 21 |
| df | 19 |
| sr | 0.192722722 |
| t | 2.814900293 |
| α | 0.05 |
| t-crit | 2.093024054 |
| p-value | 0.011058582 |
| Significant (α-=0.05) | Yes |

**Table S2.** Spearman's rank correlation coefficient of average laccase activities and consumed 2,4,5-T (a) and statistical analysis (b)

**(a)**

| Consumed [2,4,5-T] (mg/l.day) | Consumed [2,4,5-T] (Rank) | Average laccase (U/L) | Average laccase (Rank) |
| --- | --- | --- | --- |
|  |  |  |  |
| 2.6 | 10 | 2737 | 1 |
| 0.0 | 22.5 | 2115 | 10 |
| 3.2 | 9 | 2092 | 11 |
| 0.0 | 22.5 | 2552 | 3 |
| 0.0 | 22.5 | 995 | 17 |
| 0.0 | 22.5 | 1520 | 13 |
| 6.5 | 6 | 2585 | 2 |
| 4.5 | 8 | 998 | 16 |
| 0.0 | 22.5 | 1821 | 12 |
| 8.5 | 4 | 2147 | 9 |
| 7.5 | 5 | 2420 | 6 |
| 6.4 | 7 | 2247 | 8 |
| 0.0 | 22.5 | 780 | 21 |
| 0.0 | 22.5 | 999 | 15 |
| 1.2 | 12 | 925 | 19 |
| 0.2 | 13 | 377 | 27 |
| 0.0 | 22.5 | 424 | 25 |
| 1.7 | 11 | 379 | 26 |
| 16.1 | 1 | 2400 | 7 |
| 14.0 | 3 | 2467 | 4 |
| 15.2 | 2 | 2462 | 5 |
| Spearman correlation | 0.514888664 | | |

**(b)**

| Parameters | Consumed [2,4,5-T] |
| --- | --- |
| r | 0.514888664 |
| p | 0 |
| n | 21 |
| df | 19 |
| sr | 0.196668257 |
| t | 2.618056779 |
| α | 0.05 |
| t-crit | 2.093024054 |
| p-value | 0.016919881 |
| Significant (α-=0.05) | Yes |
